# Analysis of the inflammatory gene expression characteristics and immune microenvironment regulatory mechanisms in the testicular tissue of patients with non-obstructive azoospermia

**DOI:** 10.1371/journal.pone.0324948

**Published:** 2025-06-03

**Authors:** Qiang Ling, Mingqi Liu, Wei Xu, Chunhua Liao, Guijian Pang

**Affiliations:** Department of Urology, The Sixth Affiliated Hospital of Guangxi Medical University, The First People’s Hospital of Yulin, Yulin, Guangxi Zhuang Autonomous Region, China; IVF Michigan Fertility Center Bloomfield Hills: IVF Michigan Fertility Center, UNITED STATES OF AMERICA

## Abstract

**Background:**

This study aimed to deepen understanding of the molecular mechanisms and key characteristic genes of non-obstructive azoospermia (NOA).

**Methods:**

A systematic retrieval method was used to collect the mRNA expression data of NOA and obstructive azoospermia (OA) samples from the GEO database. Data preprocessing, differential gene expression screening, functional annotation, and signal pathway enrichment analysis were conducted using R software. The differences in immune microenvironment between NOA and OA samples were compared through CIBERSORT analysis. LASSO and SVM-RFE, two machine learning algorithms, were applied to select NOA-related characteristic genes. Subsequently, our investigation further identified genes differentially expressed in NOA that are associated with inflammatory responses. NOA samples were clustered based on these inflammation-related genes, while molecular features between different types were explored through pathway enrichment analysis of gene set variation analysis (GSVA). Finally, potential traditional Chinese medicine components targeting these inflammation-related genes were screened from the Chinese medicine database, followed by drug-protein docking simulations.

**Results:**

The study identified 772 DEGs mainly involved in the generation and maturation of sperm. Immune microenvironment analysis revealed significant differences in the infiltration levels of resting NK cells and activated dendritic cells between NOA and OA samples. Eight NOA-related characteristic genes were identified through LASSO and SVM-RFE algorithms. Further analysis revealed that three inflammation-related genes, namely LAMP3, PROK2, and CD14, exhibited significant differential expression in samples of NOA and OA. After clustering of these NOA samples based on the three inflammation-related DEGs, GSVA pathway enrichment analysis revealed molecular features between different NOA subtypes. Finally, potential traditional Chinese medicine components targeting these inflammation-related genes were selected.

**Conclusion:**

This study revealed the key molecular mechanisms and characteristic genes of NOA, especially the role of inflammation-related genes, providing new therapeutic targets and directions for the treatment of NOA.

## Background

Non-obstructive azoospermia (NOA) is a common type of male infertility characterized by the absence of mature sperm in the testicular tissue [1,2]. The pathogenesis of NOA is complex, involving interactions among genetic, environmental, endocrine, and immune factors. With the advancement of molecular biology and genomics, significant progress has been made in understanding the molecular mechanisms of NOA. The pathophysiology of NOA is primarily related to the process of sperm production and maturation [3]. Spermatogenesis is a highly complex and finely regulated process requiring the coordinated action of multiple genes and signaling pathways. In patients with NOA, spermatogenesis may be disrupted due to genetic defects, endocrine imbalances, or impaired immune factors [4]. Chromosomal abnormalities, gene mutations, and epigenetic changes have been confirmed to be associated with NOA [5,6]. Additionally, changes in the testicular endocrine environment, such as abnormal testosterone levels, may also affect the normal development of sperm [7]. Immune factors also play a significant role in the pathogenesis of NOA. Studies have shown that immune-mediated chronic testicular inflammation can lead to spermatogenic disorders [8]. Immune cells such as macrophages and dendritic cells have been proven to play key roles in testicular immune surveillance and inflammatory responses. Abnormal activation or dysfunction of these cells may alter the local immune environment, thereby affecting spermatogenesis [4,9].

In terms of treatment strategies, the treatment of NOA remains a challenge. Current treatments mainly focus on hormone therapy and surgical sperm extraction for assisted reproductive technologies. However, these methods are not always effective and do not address the root cause of the condition [10,11]. Therefore, a deep understanding of the molecular mechanisms of NOA, especially identifying molecular biomarkers and potential therapeutic targets closely related to the disease, is crucial for developing more effective treatment strategies.

This study aims to systematically analyze the differences in gene expression between NOA and obstructive azoospermia (OA) samples, exploring the molecular mechanisms and characteristic genes of NOA. Through high-throughput gene expression analysis and systematic bioinformatics analysis, we reveal the key molecular pathways and potential biomarkers of NOA. Additionally, we conduct an in-depth analysis of the immune microenvironment of NOA and selected NOA-related characteristic genes through machine learning methods, ultimately identifying potential therapeutic drugs for NOA. By thoroughly analyzing the molecular mechanisms and characteristic genes of NOA, we provide a new perspective on its complex pathophysiology and offer new directions for future diagnosis and treatment.

## Materials and methods

### 1.1. Data collection and preprocessing

This study conducted a systematic search of the GEO database using “non-obstructive azoospermia” as the keyword. Study data had to meet the following criteria for inclusion: (1) Data were derived from mRNA expression profiles obtained through microarray or high-throughput sequencing; (2) the studied tissue samples were from human testicular biopsies, including both NOA and OA samples; and (3) each dataset contained at least three samples of both NOA and OA. To ensure the consistency and comparability of the data, all datasets underwent log2 normalization. This study used the sva package in R software to merge datasets and remove batch effects.

### 1.2. Selection of differential expression genes (DEGs)

The selection of DEGs was performed using R software and the limma package. The study conducted differential analysis on the merged expression matrix using |log2FC| > 1 and adj. P. Val < 0.05 as the selection criteria. Visualization of DEGs was carried out using the ggplot2 and pheatmap packages in R, which included volcano plots and heatmaps.

### 1.3. Functional annotation and signal pathway enrichment analysis

Functional annotation and signal pathway enrichment analysis of DEGs were implemented using R software and the clusterProfiler package. Gene Ontology (GO) functional annotation covered biological processes (BP), cellular components (CC), and molecular functions (MF). The selection threshold for both GO and Kyoto Encyclopedia of Genes and Genomes (KEGG) pathway enrichment analysis was set at *p* < 0.05. Additionally, to explore the role of DEGs in the pathogenesis of NOA, the study obtained gene sets from the gene set enrichment analysis (GSEA) official website, including c5.go.Hs.symbols.gmt and c2.cp.kegg.Hs.symbols.gmt, and conducted GSEA on DEGs. The Metascape online database was also utilized to identify diseases related to DEGs.

### 1.4. Immune microenvironment analysis

An in-depth analysis was conducted of the differences in immune microenvironment between NOA and OA. Immune cell infiltration analysis was performed using the CIBERSORT algorithm, calculating the immune infiltration scores for each sample and comparing the levels of immune cell infiltration between NOA and OA samples.

### 1.5. Selection of NOA characteristic genes

The study employed the least absolute shrinkage and selection operator (LASSO) and support vector machine recursive feature elimination (SVM-RFE) algorithms to select NOA-related characteristic genes. These algorithms independently processed DEGs, yielding two sets of genes, and the intersection of these sets determined the final NOA characteristic genes. Subsequently, the study analyzed the differences in the expression of these characteristic genes between NOA and OA samples and their association with immune cell infiltration levels.

### 1.6. Identification of inflammation-related genes differentially expressed in NOA

To explore the role of inflammation in the development and progression of NOA, the study integrated a wide range of literature data and selected reported inflammation-related genes. These genes were then cross-compared with the DEGs of NOA to identify inflammation-related genes associated with NOA. The relationships among these genes were analyzed and visualized using the corrplot and circlize packages in R.

### 1.7. Sample clustering and typing

Based on the inflammation-related genes differentially expressed in NOA, the study used R software and the ConsensusClusterPlus package to perform clustering and typing of the NOA samples. The limma, pheatmap, reshape2, and ggpubr packages were used to analyze the typing results, constructing box plots and heatmaps of subtypes. To compare the differences and similarities within different NOA subtypes, the study employed the limma and ggplot2 packages for analysis and created principal component analysis (PCA) scatter plots. Additionally, the study compared the differences in expression of inflammation-related DEGs between different NOA subtypes.

### 1.8. Post-typing gene set variation analysis (GSVA)

Based on the typing results of inflammation-related DEGs, GSVA pathway enrichment analysis was conducted on NOA expression data. Pathways with an adjusted *p*-value less than 0.05, either upregulated or downregulated, were selected and have been visually presented in bar graphs.

### 1.9. Screening of Traditional Chinese Medicine components for inflammation-related genes differentially expressed in NOA

The study selected potential biologically active traditional Chinese medicine components targeting inflammation-related genes differentially expressed in NOA from the HERB database. Structural information of Chinese medicine components and target proteins was obtained from the PubChem and RCSB Protein Data Bank databases. Drug molecule–protein docking simulations were performed using ChemBio3D Ultra, PyMOL, and AutoDock Tools software to assess the potential binding of Chinese medicine components to target proteins.

### 1.10. Ethics approval

Ethical approval was not required because the study used publicly available data, adhering to the ethical standards of the First People’s Hospital of Yulin.

## Results

### 2.1. Differential gene expression in NOA

**Fig 1:**
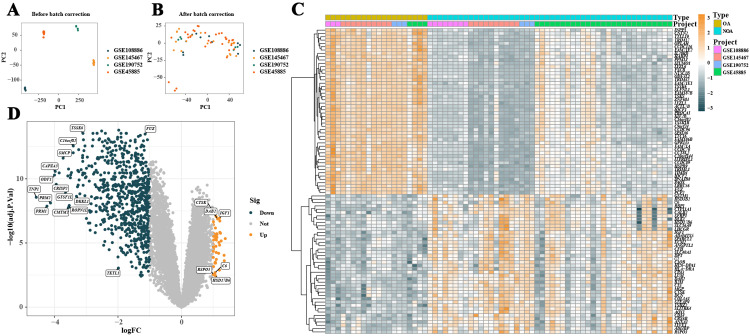
Analysis of gene expression differences between non-obstructive azoospermia (NOA) and obstructive azoospermia (OA) samples. **A** shows the principal component analysis before batch effect correction. **B** illustrates the principal component analysis after batch effect correction. **C** presents a heatmap of differentially expressed genes, visualizing the top 50 upregulated and downregulated genes between NOA and OA samples. **D** is a volcano plot showing the distribution of all differentially expressed genes between NOA and OA samples.

The study identified the following four NOA datasets: GSE145467, GSE108886, GSE190752, and GSE45885. The samples in each dataset originated from human testicular biopsy tissues and included three or more samples of both NOA and OA. After merging these datasets and removing batch effects, a comprehensive expression dataset of 14,975 genes was obtained (Fig 1A and B). Further differential expression analysis revealed that 772 genes exhibited significant differential expression between NOA and OA samples (Fig 1C–D).

### 2.2. Functional and pathway enrichment analysis of DEGs

**Fig 2:**
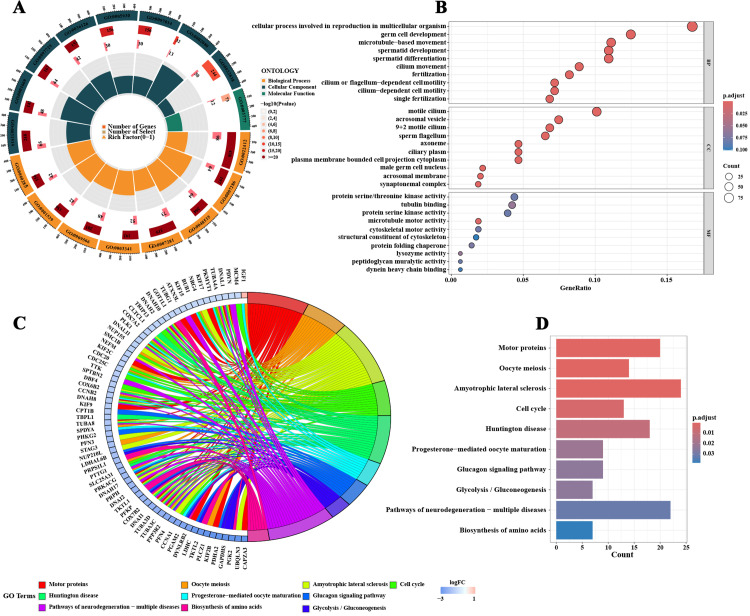
Functional and pathway enrichment analysis of differentially expressed genes in non-obstructive azoospermia. **A and B** display the results of the gene ontology analysis, highlighting the involvement of differentially expressed genes in key biological processes such as meiosis, the development of spermatogonia, sperm motility, and structure formation. **C and D** show the results of the Kyoto Encyclopedia of Genes and Genomes pathway analysis, revealing the significant functions of these genes in pathways such as motor proteins, glucagon signaling, and amino acid biosynthesis.

**Fig 3:**
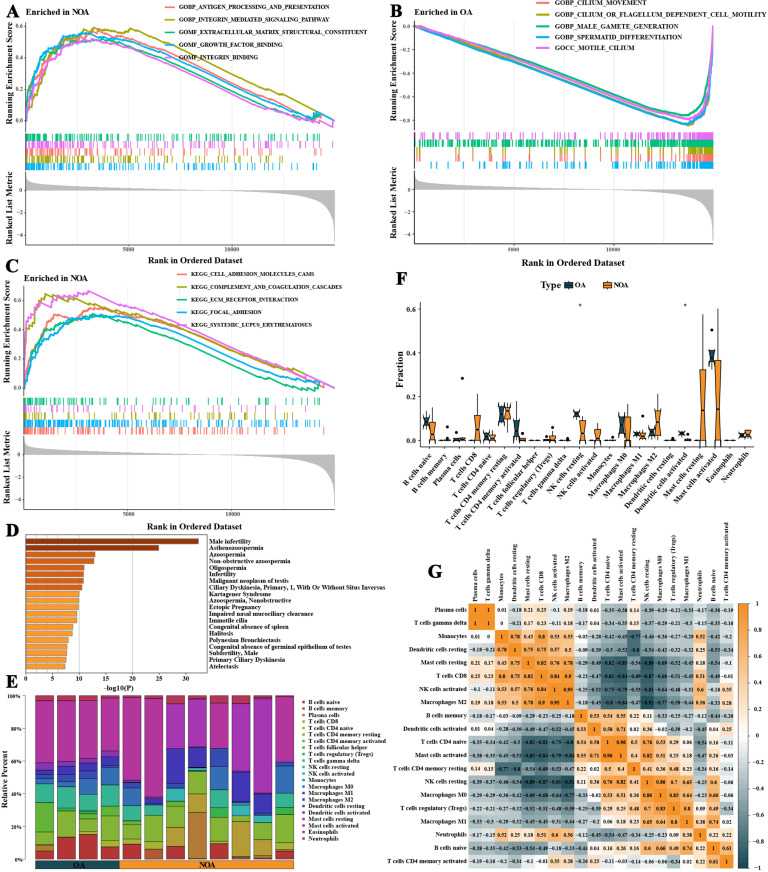
Gene set enrichment analysis and immune microenvironment assessment of differentially expressed genes in non-obstructive azoospermia (NOA). **A–C** depict the results of gene set enrichment analysis, showing biological processes potentially promoted by upregulated differentially expressed genes in NOA, including CDC42 protein signaling transduction, the production of transforming growth factor-beta, and the VEGF receptor signaling pathway. **D** illustrates the results of the Metascape database analysis, linking these genes to diseases such as primary ciliary dyskinesia and male infertility. **E** shows the results of the CIBERSORT analysis, depicting the proportions of immune cell infiltration in NOA and OA samples from the GSE145467 dataset. **F** highlights the differences in immune cell proportions between NOA and OA samples, suggesting specific immune regulatory mechanisms in NOA. **G** presents the correlation analysis of immune cell infiltration levels.

The functional and biological process involvement of these DEGs was deeply explored through GO, KEGG, and GSEA analyses. GO analysis indicated that these genes were mainly involved in processes such as meiosis, development and differentiation of spermatogonia, and sperm motility and activation and were closely related to the formation of sperm structures such as the flagella, sperm plasma membrane, and sperm head (Fig 2A and B). KEGG pathway analysis revealed the significant roles of these genes in pathways such as motor proteins, the glucagon signaling pathway, and amino acid biosynthesis (Fig 2C and D). GSEA also suggested that upregulated DEGs in NOA can promote biological processes such as CDC42 protein signaling transduction, the production of transforming growth factor-beta, the VEGF receptor signaling pathway, binding of insulin-like growth factor I, the MAPK signaling pathway, and the Toll-like receptor (TLR) signaling pathway. Conversely, they might inhibit processes such as meiosis, sperm DNA condensation, spermatid differentiation, and sperm motility and vitality and affect the formation of structures such as the sperm plasma membrane, sperm head, acrosomal vesicle, and sperm midpiece (Fig 3A–C; S1 and S2 Files). Additionally, analysis using the Metascape database revealed that these DEGs were related to diseases such as primary ciliary dyskinesia, male infertility, immotile cilia, Kartagener syndrome, congenital absence of germinal epithelium in the testis, azoospermia, asthenozoospermia, infertility, and non-obstructive azoospermia (Fig 3 D).

### 2.3. Immune microenvironment differences between NOA and OA

CIBERSORT analysis of the immune microenvironment in the GSE145467 dataset for NOA and OA samples calculated the proportions of 22 types of immune cells in each sample (Fig 3E). Subsequent t-tests comparing immune cells between NOA and OA groups showed that NOA samples had a lower proportion of resting NK cells and activated dendritic cells, suggesting that NOA might be associated with specific immune regulatory mechanisms (Fig 3 F). Fig 3G shows the correlations in the degree of immune cell infiltration.

### 2.4. Identification of disease characteristic genes for NOA

**Fig 4:**
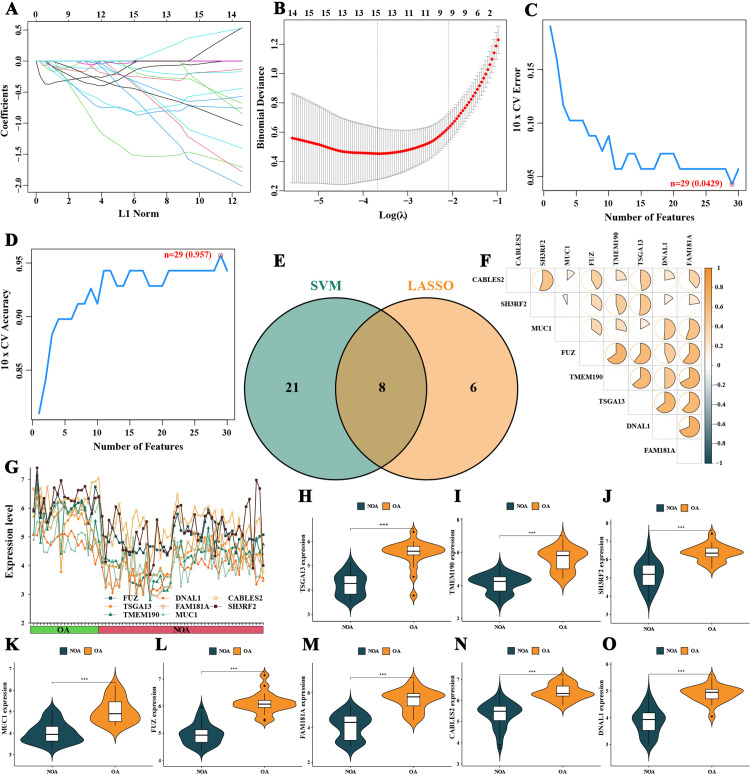
Selection and analysis of characteristic genes for non-obstructive azoospermia (NOA). **A and B** show the results of the least absolute shrinkage and selection operator (LASSO) algorithm. **C and D** depict the results of the support vector machine recursive feature elimination (SVM-RFE) algorithm. **E** analyzes the intersection of the results from both algorithms, identifying eight key NOA characteristic genes. **F–O** analyze the expression trends of these genes, showing positive correlations in gene expression and down-regulation in NOA samples.

**Fig 5:**
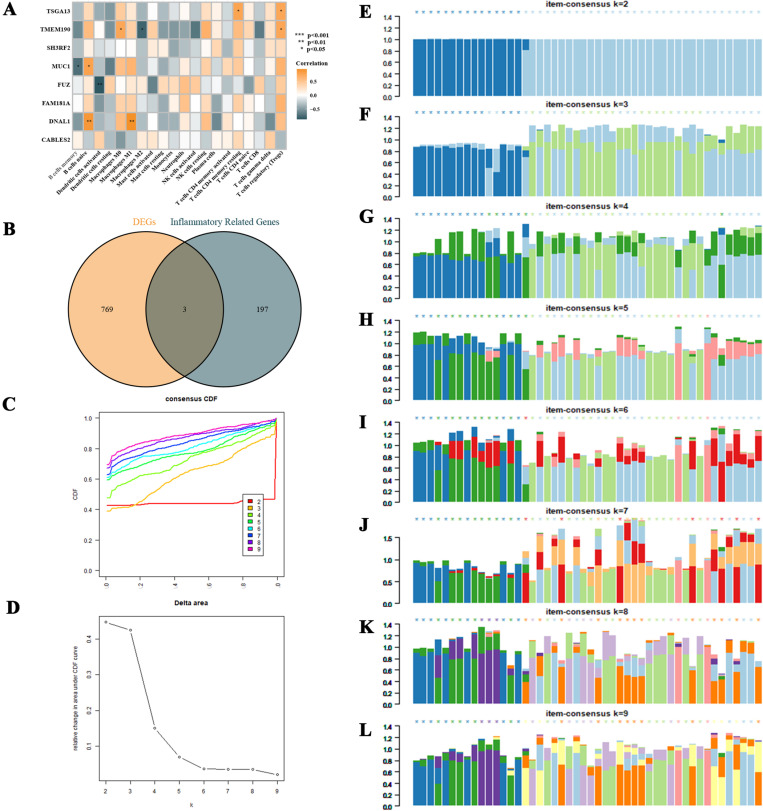
Analysis of the correlation between characteristic genes and immune cell infiltration in non-obstructive azoospermia (NOA) and clustering typing of NOA samples based on inflammation-related genes. **A** analyzes the correlation between characteristic genes and levels of immune cell infiltration. **B** selects inflammation-related genes differentially expressed in NOA through literature research and analysis of differentially expressed genes. **C–L** demonstrate clustering typing of NOA samples based on inflammation-related genes differentially expressed in NOA, showing the highest differentiation, stability, and consistency scores of subtypes when the k-value is set to 2.

To identify key characteristic genes between NOA and OA, two machine learning algorithms, LASSO and SVM-RFE, were employed. The LASSO algorithm identified 14 characteristic genes, including CCDC116, FUZ, and INPP1 (Fig 4A and B), while the SVM-RFE algorithm identified 29 characteristic genes, including FUZ, GLT1D1, and GGTLC1 (Fig 4C and D). The intersection of the results from both algorithms identified eight NOA characteristic genes, including FUZ, TSGA13, TMEM190, DNAL1, FAM181A, MUC1, CABLES2, and SH3RF2 (Fig 4E). These genes showed an down-regulated expression trend in NOA samples (Fig 4G–O) and exhibited positive correlations in gene expression (Fig 4F). Fig 5A displayed the correlation between characteristic genes and levels of immune cell infiltration.

### 2.5. Analysis of NOA sample clustering and typing

**Fig 6:**
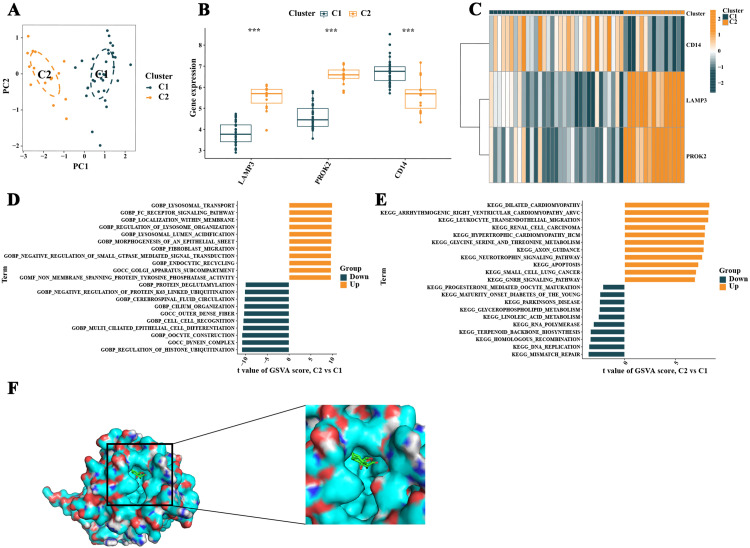
Subtype analysis of non-obstructive azoospermia (NOA) samples and screening of traditional Chinese medicine components targeting inflammation-related genes differentially expressed in NOA. **A** shows a PCA scatter plot illustrating significant differences between two NOA subtypes based on inflammation-related genes differentially expressed in NOA. **B** and **C** display the expression differences of inflammation-related genes in different subtypes. **D** and **E** present the results of an analysis of post-typing gene set variation, revealing up-regulated and down-regulated biological pathways in different subtypes. **F** shows the molecular docking simulation results of artemisinin with the CD14 protein, including a visualization of the docking model with the highest binding affinity.

Through extensive literature research, the study compiled 200 genes related to inflammation and cross-compared them with the 772 DEGs of NOA, ultimately selecting the following three inflammation-related genes differentially expressed in NOA: LAMP3, PROK2, and CD14 (Fig 5B). Based on these genes, clustering typing of NOA samples was performed in the merged dataset. The study found that when the k-value was set to 2, the consensus clustering matrix had the highest differentiation, the number of clusters was most stable, and the consistency scores of each subtype were highest (Fig 5C–L). The PCA scatter plot also showed significant differences between the two subtypes (Fig 6A). Fig 6B and C displayed the expression differences of inflammation-related DEGs in different subtypes.

### 2.6. Post-Typing GSVA pathway enrichment analysis

The GSVA pathway enrichment analysis conducted after typing based on inflammation-related DEGs revealed that the upregulated pathways mainly involved the FC receptor signaling pathway, morphogenesis of an epithelial sheet, fibroblast migration, the GNRH signaling pathway, axon guidance, and apoptosis. The downregulated pathways included the dynein complex, multi-ciliated epithelial cell differentiation, negative regulation of protein K63–linked ubiquitination, protein deglutamylation, regulation of histone ubiquitination, mismatch repair, DNA replication, terpenoid backbone biosynthesis, glycerophospholipid metabolism, linoleic acid metabolism, maturity onset diabetes of the young, and Parkinson’s disease (Fig 6D and E).

### 2.7. Screening of traditional chinese medicine components for inflammation-related genes differentially expressed in NOA

Finally, targeting the three inflammation-related DEGs, potential targeted components of traditional Chinese medicine were screened from the HERB database. These included potential targeting components for CD14, such as artemisinin and fisetin; for PROK2, such as 2n-hexadecanoic acid, acetylcholine, cetylic acid, cis-9-octadecenoic acid, and cis-oleic acid; and for LAMP3, such as 17-β-estradiol, 3,4-benzopyrene, and di(2-ethylhexyl)phthalate. Notably, the expression of CD14 was significantly up-regulated in NOA. And artemisinin, identified as a potential drug for CD14, has been widely reported for its anti-inflammatory and immune-regulatory effects. The study utilized ChemBio3D Ultra, PyMOL, and AutoDock Tools software for molecular docking simulations of artemisinin with the CD14 protein. The specific docking results are detailed in Table 1. The first docking mode (Mode 1) exhibited the highest binding affinity (−7.3 kcal/mol) and an RMSD value of 0, suggesting it might be the optimal docking posture. Therefore, the Mode 1 model was selected for visual representation (Fig 6F). This analysis provides important clues for further drug development.

**Table 1:** Summary of drug molecule–protein docking results using AutoDock Tools software.

Mode	Affinity (kcal/mol)	rmsd l.b.	rmsd u.b.
1	−7.3	0	0
2	−7.2	8.055	9.874
3	−7.1	6.691	8.478
4	−6.9	7.436	9.438
5	−6.9	8.426	9.452
6	−6.9	4.303	7.194
7	−6.8	51.494	52.4
8	−6.8	6.853	8.165
9	−6.7	2.148	4.208

## Discussion

In this study, we systematically explored the molecular mechanisms and characteristic genes of NOA to deepen our understanding of its pathogenesis and explore potential therapeutic targets. By comparing NOA and OA samples, the study revealed a series of DEGs and conducted functional annotation and signal pathway enrichment analysis for these genes. Additionally, we conducted an in-depth analysis of the immune microenvironment of NOA and used machine learning methods to select characteristic genes closely related to NOA. Subsequently, We identified several inflammation-related DEGs and analyzed their expression patterns in NOA samples. The study also screened potential traditional Chinese medicine components targeting these inflammation-related DEGs. This research not only provides new insights into the pathophysiological mechanisms of NOA but also offers potential molecular targets for subsequent clinical treatment strategies.

First, by systematically analyzing the gene expression differences between NOA and OA samples, the study identified 772 significant DEGs. These genes are primarily involved in the normal occurrence and maturation of sperm, significantly affecting sperm motility and fertilization capacity. For instance, genes such as RNF17, SYCP3, SYCE1, STAG3, TNP1, and TNP2, which have crucial roles in spermatogenesis, were downregulated in NOA. RNF17 is a key component of male germ cell differentiation, and its absence leads to infertility in male mice, with germ cells ceasing development at the round spermatid stage and failing to produce sperm [12]. SYCP3 and SYCE1, as important components of the synaptonemal complex (SC), facilitate homologous chromosome pairing and recombination during meiosis [13,14]. Mutations or functional loss of SYCP3 and SYCE1 can disrupt the meiotic process, leading to abnormal chromosome pairing and recombination, thereby affecting normal sperm development and maturation [13,15,16]. STAG3 regulates the separation of sister chromatids during meiosis [17–19], and its absence disrupts the cohesion of sister chromatids, leading to shortened or absent axial elements and SC during meiosis, causing spermatocytes and oocytes to cease meiosis [20–24]. TNP1 and TNP2 are associated with chromatin remodeling during spermatogenesis, crucial for sperm head formation and DNA stability, and their downregulation may lead to abnormal sperm morphology [25–28]. Through GO, KEGG, and GSEA analyses, we found that these DEGs participate in multiple key biological processes and signaling pathways related to spermatogenesis, such as inhibiting meiosis, sperm DNA condensation, spermatid differentiation, and sperm motility and vitality and affecting the formation of structures such as the sperm plasma membrane, sperm head, acrosomal vesicle, and sperm midpiece. These findings further confirm that impaired sperm formation and maturation capacity may be key factors in the pathogenesis of NOA.

In terms of the immune microenvironment analysis, existing studies have shown that immune cells play a significant role in the male reproductive system and that immune-mediated chronic testicular inflammation is one of the main causes of NOA [29]. Zheng et al., through single-cell RNA sequencing and analysis of the Human Protein Atlas database, confirmed that macrophages are the largest immune cell group in normal testes, exhibiting a state of reduced inflammatory response by expressing high levels of tolerance proteins and reducing the expression of TLR pathway–related genes. In contrast, the immune microenvironment in NOA tissues is characterized by an activated immune system and a pro-inflammatory state. Further analysis has suggested that the number of M1 and M2 macrophages in NOA tissues is significantly higher than in normal testes and that the number of macrophages, including M1 and M2 types, is negatively correlated with the spermatogenesis score in NOA patients [30]. Our study revealed the composition of specific types of immune cells in NOA and OA samples and found that NOA samples had a lower proportion of resting NK cells and activated dendritic cells than OA samples. Dendritic cells in the epididymis regulate the complex interplay between immune tolerance and activation, participating in establishing and maintaining immune tolerance to mature sperm expressing new self-antigens, thereby affecting male fertility [31,32]. These findings suggest that NOA may be associated with unique immune regulatory mechanisms, providing a basis for further research on the immune regulation mechanisms of NOA.

Furthermore, using LASSO and SVM-RFE machine learning algorithms, the study successfully identified eight characteristic genes closely related to NOA. These genes showed a significant down-regulation trend in NOA samples, suggesting they may play important roles in the pathogenesis of NOA. Additionally, we paid special attention to inflammation-related DEGs. Studies have shown that chronic inflammation leads to increased oxidative stress, elevated levels of reactive oxygen species and pro-inflammatory cytokines, and impaired steroidogenesis [8]. Moreover, chronic inflammation may lead to the influx of pro-inflammatory macrophages into the testes, affecting the metabolism of immune cells and altering the normal immunosuppressive microenvironment, leading to germ cell apoptosis and disrupting the spermatogenesis process [4,9]. Our study found that LAMP3, PROK2, and CD14, three inflammation-related DEGs, showed significant expression differences in NOA samples, suggesting they play key roles in the inflammatory process of NOA. This finding provides new clues for further research into the role of inflammation in the pathogenesis of NOA.

Research has shown that traditional Chinese medicine can regulate the hypothalamic–pituitary–testicular axis, enhance the function of supporting and interstitial cells, reduce the DNA fragmentation index, prevent oxidative stress, alleviate inflammation, and regulate the proliferation and apoptosis of germ cells, thereby improving male infertility [33–36]. Therefore, this study also screened potential traditional Chinese medicine components targeting inflammation-related DEGs, providing some potential therapeutic targets for the treatment of NOA as well as a foundation for future drug development and clinical application. Our study, by targeting three inflammation-related DEGs, screened potential targeted components of traditional Chinese medicine in the HERB database. Artemisinin, derived from the artemisia plant and a widely used antimalarial drug, has recently attracted attention for its anti-inflammatory and immune-regulatory effects. Studies have shown that artemisinin exhibits potential therapeutic effects in various disease states by regulating multiple immune cells and inflammatory pathways. Shi et al.’s research revealed artemisinin’s ability to alleviate neuroinflammation in the central nervous system, with mechanisms including regulating inflammatory processes and anti-oxidative stress, inhibiting systemic inflammation, alleviating intestinal inflammation, and preventing Aβ accumulation [37]. Qiao et al. found that myeloid differentiation factor 2 (MD-2) may be a potential target of artemisinin. MD-2, in combination with Toll-like receptor 4 (TLR4), participates in immune responses. The binding of artemisinin and its derivatives to MD-2 reduces the stability of the TLR4/MD-2 complex, thereby exerting anti-inflammatory and immune-regulatory effects [38]. Additionally, artemisinin regulates immune responses by affecting the activation of regulatory T cells, downregulating the expression of pro-inflammatory factors, inducing macrophage polarization toward the M2 phenotype, inhibiting epithelial–mesenchymal transition, and balancing Th1/Th2 responses [39,40]. In terms of inflammatory pathways, Wang et al.’s research confirmed that artemisinin regulates the NF-κB and MAPK signaling pathways and inhibits inflammation-driven lymphangiogenesis by affecting the VEGF-C/VEGFR-3 pathway, thereby suppressing inflammatory responses [41,42]. Therefore, the effects of artemisinin may make it a potential therapeutic agent for treating inflammation-related diseases. Notably, the expression of CD14 was significantly up-regulated in NOA. Through drug molecule-protein docking simulations, our study confirmed that artemisinin could form a stable binding with CD14, suggesting that artemisinin will become a potential therapeutic drug for NOA by inhibiting inflammatory responses and regulating immunity. However, the application of artemisinin in the treatment of NOA requires further research to verify its efficacy and safety.

In summary, our study provides new insights into the molecular mechanisms and immune characteristics of NOA and opens up new avenues for future research and treatment strategies through the discovery of inflammation-related genes differentially expressed in NOA. However, it also has some limitations. For example, our analysis mainly relied on data from public databases that may have had certain biases and limitations. Therefore, future research should include more extensive and in-depth clinical sample analyses to verify and expand on our findings. Additionally, the inflammation-related genes and potential drug targets we identified require further experimental validation and clinical trials to assess their practical application value in the treatment of NOA. In conclusion, our study provides a new perspective for understanding the complex biology of NOA and offers new directions for future diagnosis and treatment.

## Conclusions

This study revealed the key molecular mechanisms and characteristic genes of NOA, especially the role of inflammation-related genes, providing new therapeutic targets and directions for the treatment of NOA.

## Supporting information

S1 File Biological process, cellular component, and molecular function enrichment results based on GSEA using the c5.go.Hs.symbols.gmt gene set. (TXT)

S2 File Pathway enrichment results based on GSEA using the c2.cp.kegg.Hs.symbols.gmt gene set. (TXT)
